# Nicotinamide riboside enhances chondrogenic differentiation of human adipose stem cells via inducing SIRT1-mediated deacetylation of SMAD7 and activation of TGF-β/SMAD2/3 pathway

**DOI:** 10.1186/s13287-026-05226-8

**Published:** 2026-08-08

**Authors:** Yichi Xu, Jun Shu, Youbai Chen, Haimiao Wang, Ruoxu Zhang, Muzi Chen, Ran Tao

**Affiliations:** 1 Department of Plastic and Reconstructive Surgery, The First Medical Center of Chinese PLA General Hospital, Beijing, 100853 China; 2 Senior Department of Burns and Plastic Surgery, The Fourth Medical Center of Chinese PLA General Hospital, Beijing, 100048 China

**Keywords:** Nicotinamide riboside, Chondrogenic differentiation, SIRT1, SMAD7, SMAD2/3

## Abstract

Mesenchymal stem cells (MSCs) are widely regarded as a promising candidate for cartilage regeneration, and MSC-based therapies have demonstrated therapeutic potential in promoting cartilage repair. Nevertheless, the chondrogenic differentiation efficacy still requires further improvement. This study identified that Nicotinamide Riboside, a precursor of nicotinamide adenine dinucleotide (NAD⁺), significantly enhances chondrogenic differentiation efficiency of human adipose-derived stem cells (hADSCs), primary by activating the TGF-β/SMAD2/3 signaling pathway. Mechanistically, we demonstrated that NR upregulated the expression of SIRT1 and enhanced SIRT1 activity during the induction of chondrogenesis, thereby promoting the deacetylation and subsequent degradation of SMAD7. NR-mediated reduction of SMAD7 results in enhanced phosphorylation of SMAD2/3 and activation of the TGF-β/SMAD2/3 signaling pathway. SIRT1-mediated deacetylation of SMAD7 is a key factor in NR-enhanced chondrogenic differentiation, as disruption of the interaction between SIRT1 and SMAD7 significantly abolishes the promotive effect of NR on chondrogenesis. Collectively, our study demonstrates that NR enhanced chondrogenic differentiation of hADSCs by regulating SIRT1-mediated deacetylation of SMAD7 and activation of TGF-β/SMAD2/3 pathway. NR may therefore possess prospects as a promising supplement for MSC-based cartilage regeneration and repair therapies.

**Supplementary Information:**

The online version contains supplementary material available at https://doi.org/10.1186/s13287-026-05226-8.

## Introduction

Human cartilage tissue demonstrates a relatively limited healing potential and poses significant difficulties to repair itself when it is damaged [1]. Effective approaches for cartilage regeneration are critically needed to facilitate cartilage repair, treatment of cartilage defects and cartilage-associated diseases such as Osteoarthritis [2]. Mesenchymal stem cells (MSCs) are considered an ideal option for cartilage regeneration. This is because they can differentiate into chondrocytes, possess a highly regulated self-renewal capacity, and secrete bioactive factors that promote tissue healing. MSC-based therapies have shown beneficial effects in a broad range of preclinical trials for cartilage repair or reconstruction [3–6]. However, at present, there are still several challenges related to clinical therapies for cartilage regeneration using MSCs, including differentiation efficiency.

Nicotinamide Riboside (NR), belongs to the vitamin B3 family, is one of the precursors of nicotinamide adenine dinucleotide (NAD⁺) [7, 8]. NAD^+^ is a crucial molecule in human life and health, as it participates in numerous biological processes, including energy metabolism, cell signaling and mitochondrial homeostasis. NR, as a NAD^+^ booster, has been attracted widespread attention due to its beneficial effects in metabolism disorders, aging, fibrosis, neurodegenerative diseases and inflammation related diseases [9–12]. In addition, several studies have shown that the oral administration of NAD^+^ precursors, including NR and NMN, is safe and leads to a significant increase in NAD^+^ levels in humans [13, 14]. Consequently, NR supplementation has been increasingly regarded as an effective strategy to enhance intracellular NAD^+^ concentrations to benefit human health. However, research on the effects of NR in cartilage-related diseases remains limited. A recent study has identified reduced NAD^+^ levels in both human and murine osteoarthritis (OA), and supplementation with NAD^+^ precursors has been shown to attenuate cartilage disruption in aging murine and surgical rat models of OA [15]. Their study suggested the potential of NAD⁺ precursor treatment in protecting against cartilage degeneration. Nevertheless, the underlying mechanism remains unclear, and the effect of NR on cartilage regeneration, particularly its influence on the chondrogenic differentiation of MSCs, is still largely unknown.

TGF-β/SMAD2/3 pathway plays a critical role in chondrocyte differentiation and the maintenance of cartilage matrix [16]. Upon binding to transforming growth factor receptor, SMAD2/3 are phosphorylated. The phosphorylation of SMAD2/3 is a critical step in activating the TGF-β/SMAD2/3 signaling pathway and facilitating its nuclear translocation, thereby promoting the expression of chondrocyte-related genes such as COL2A1 and SOX9 [17], which are essential for cartilage formation [18]. Therefore, enhancing the phosphorylation of SMAD2/3 represents an effective strategy to activate the TGF-β/SMAD2/3 signaling pathway and facilitate chondrocyte differentiation.

SMAD7 has been recognized as an intracellular inhibitor of the TGF-β/SMAD2/3 signaling pathway, acting through competitive binding to the TGF-β receptor complex, which effectively prevents the interaction between SMAD2/3 and the TGF-β receptor [19, 20]. Furthermore, SMAD7 interacts with the activated TGF-β receptor more stable than SMAD2/3, thereby interfering with the association, phosphorylation, and activation of SMAD2/3 [21]. Previous studies have demonstrated that abnormal SMAD7 expression is associated with various diseases through the regulation of the TGF-β signaling pathway, including skeletal muscle atrophy and fibrosis, kidney diseases, fibroblast activation, and hypertrophic scar formation [22–24]. Therefore, SMAD7 may play an important regulatory role in the chondrogenesis related to the TGF pathway. Therefore, SMAD7 may play an important role in TGF-β/SMAD2/3-mediated chondrogenic differentiation.

In this study, we evaluated the role and potential mechanism of NR on chondrogenic differentiation of human adipose stem cells (hADSCs). We demonstrated that NR has a significant positive regulatory effect on chondrogenic differentiation. SIRT1-mediated deacetylation of SMAD7 may contribute to NR-promoted chondrogenic differentiation.

## Materials and methods

### Isolation and culture of hADSCs

Human subcutaneous adipose tissues were collected from nine independent female donors aged 18–35 years who underwent abdominal liposuction surgery. Collection and use of Human subcutaneous adipose tissues were approved by the Institutional Review Board of the First Medical Center of PLA General Hospital (Approval number: EAC-IACUC-2025F74-069). The hADSCs were extracted from human fresh lipoaspirate. The adipose tissue was digested with 0.05% collagenase type II (Sigma, U.S.A.) for 50 min at 37 ^◦^C, vortexed, and centrifuged at1200 rpm for 10 min, and the stromal vascular fraction (SVF) obtained. Afterward, the pellet (SVF), containing hADSCs, was resuspended with DMEM medium (Gibco; Thermo Fisher Scientific, Inc.; cat. no. 11965092), supplemented with 10% fetal bovine serum (Gibco; Thermo Fisher Scientific, Inc; cat. no. 10099141), and 100 units/mL penicillin, and 100 µg/mL streptomycin (Hyclone; Cytiva). Culture medium was changed 48 h after initial plating and floating cells were removed. The culture medium was changed every two days until an 90% confluence was reached. All experiments were performed using hADSCs derived from nine donors, with three independent biological replicates set for each condition.

### Chondrogenic differentiation

hADSCs were trypsinized and reseeding in at a density of 2 × 10^7^ cells/mL in Chondrogenic Differentiation induced Medium from StemPro™ Chondrogenesis Differentiation Kit (Thermo Fisher, USA). 15µL droplet were seeded in each interior well of a 24-well plate and allowed to adhere for 2 h. When the cells adhered, 500 µL medium with or without NR (200µM) was added. The medium was changed every two days. After 2 weeks of chondrogenic induction, the pellets were harvested and Safranin O and Alcian blue staining were performed.

### Quantitative real time-PCR

Cells were harvested, and TRIzol^®^ Reagent (Invitrogen, USA) was employed to extract total RNA following manufacturer’s instructions. Reverse transcription was performed using the HiScript III 1st Strand cDNA Synthesis Kit (Vazyme Biotech Co., Nanjing, China) according to the manufacturer’s protocol. Gene expression was evaluated by qPCR using the ChamQ SYBR Color qPCR Master Mix (Vazyme Biotech Co., Nanjing, China) and run on the ABI STEPONE system (Applied Biosystems, Foster City, CA, USA). The thermocycling protocol included an initial denaturation step at 95 °C for 3 min, followed by 40 cycles of denaturation at 95 °C for 15 s, annealing at 60 °C for 15 s and extension at 72 °C for 30 s. The 2-ΔΔCq method was calculated to analyze the relative changes in gene expression according to the previous reported method (Livak and Schmittgen, 2001). β-Actin was used as an internal control. Primers used in this study are listed in supplementary Table S1.

### Western blot analysis

The cells were lysed with RIPA buffer (Beyotime Institute of Biotechnology) supplemented with a protease and phosphatase inhibitor cocktail (Beyotime Institute of Biotechnology). The proteins were separated by SDS-PAGE gels and subsequently transferred onto polyvinyl difluoride (PVDF) membranes (Labselect; cat. no. TM-PVDF-R-45). The membranes were blocked with 5% skimmed milk (Difco; BD Biosciences) and immunoblotted with primary antibodies at 4˚C overnight, followed by incubation with secondary antibodies for 2 h at room temperature. Luminescence was detected using the Beyo ECL Moon super sensitivity detection kit (Beyotime Institute of Biotechnology) according to the manufacturer’s protocol. The following antibodies were used: Anti-COL2A1 antibody( Proteintech 28459-1-AP), SOX9 ( Proteintech 67439-1-Ig), ACAN ( Proteintech 68350-1-Ig), SMAD2 ( Proteintech 12570-1-AP), SMAD3 ( Proteintech 66516-1-Ig), SMAD7 ( Proteintech 25840-1-AP), p-SMAD2 (Proteintech 80427-2-RR), p-SMAD3, (Cell signal #9520S), p-SMAD2/3 (Invitrogen PA5-110155), SIRT1(Proteintech 13161-1-AP), Acty-SMAD2 (Immunoway YK0167), Acty-SMAD3 (Abmart PU914561), Acty-SMAD7 (Immunoway YK0168),GAPDH (Proteintech 60004-1-Ig).

### Immunofluorescence

For immunofluorescence staining, cells were cultured on NUNC Lab-Tek II chamber slides (Fisher Scientific, 12-565-2) for 48 h before experimental treatment. Cells were fixed with 4% paraformaldehyde-PBS, rinsed twice with PBS, permeabilized with 0.1% Triton X-100/PBS, blocked in 3% goat serum in 0.1% (v/v) Triton X-100/PBS, and stained with the indicated primary antibodies overnight at 4 °C followed by incubation with fluorescent conjugated secondary antibodies. and then mounted with DAPI-containing mounting medium (Beyotime, P0131). Fluorescence signals were captured at room temperature with a confocal microscope (Leica DMI6000B-TCS-SP8 confocal system, Germany). Images were collected and analyzed by LAS-AF software (Leica Ltd.) and ImageJ (National Institutes of Health) software. The degree of protein colocalization was quantified in ≥ 100 cells from three independent experiments and analyzed using ImageJ. The following antibodies were used: Anti-COL2A1 Antibody (Proteintech 28459-1-AP ), Anti-SOX9 Antibody(Proteintech 55152-1-AP). SMAD7 (Proteintech 25840-1-AP), p-SMAD2/3 (Invitrogen PA5-110155).

### Statistical analyses

The data are expressed as the means ± SDs from a minimum of three independent biological replicate experiments. Technical replicates in triplicate were averaged before statistical analysis. Statistical analysis was conducted using GraphPad Prism 5.0 software (GraphPad Software, Inc., La Jolla, CA, USA). Normality of the data was assessed using the Shapiro-Wilk and Kolmogorov-Smirnov tests. For comparisons between two groups, a two-tailed paired Student’s t-test was employed. Comparisons among multiple groups were performed using one-way or two-way ANOVA followed by Tukey’s post-hoc test. Statistical significance was regarded as *P < 0.05 or **P < 0.01, ***P < 0.001.

## Results

### NR enhances chondrogenic differentiation of hADSCs

**Fig. 1:**
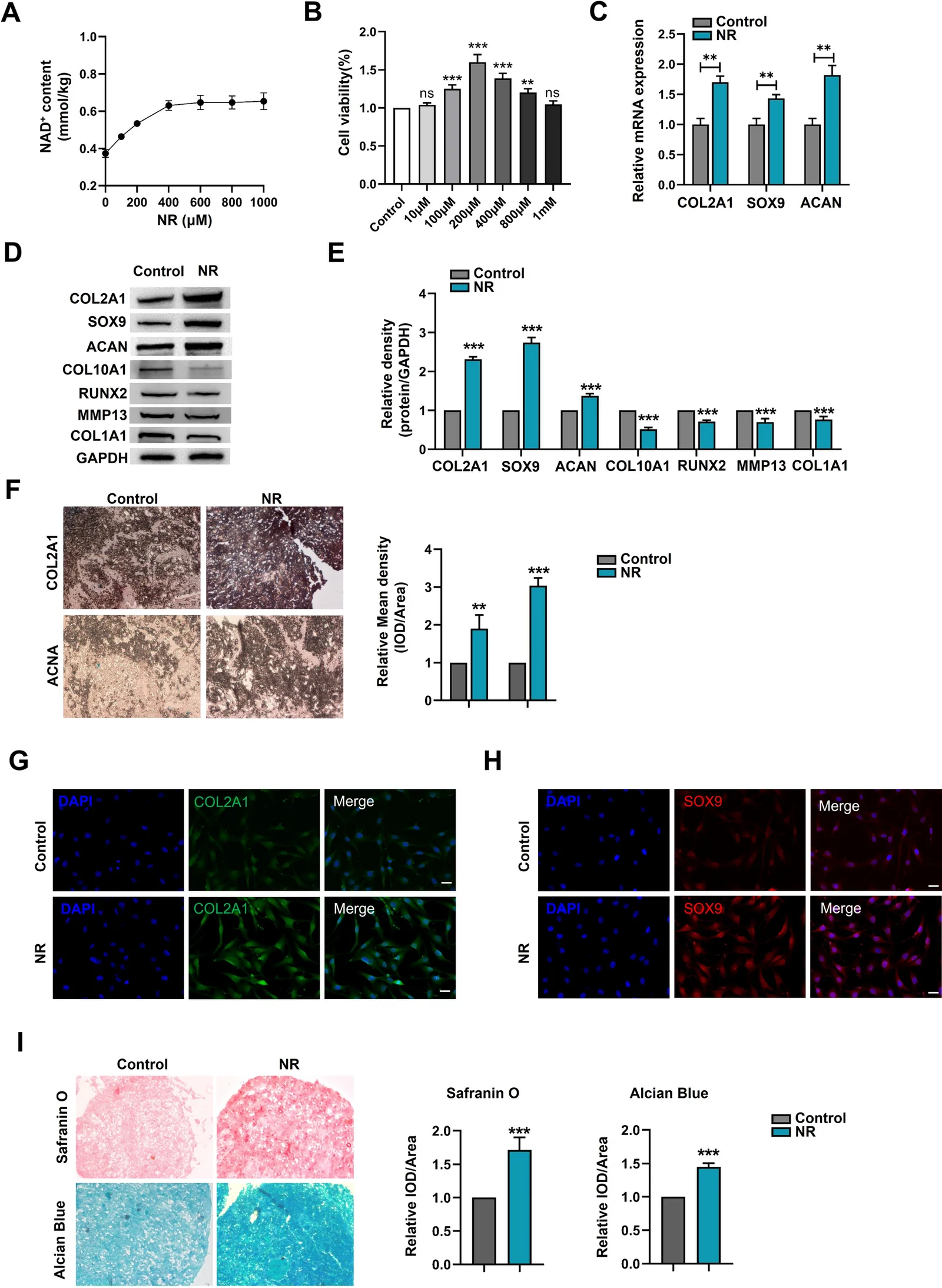
NR enhances chondrogenic differentiation of hADSCs. **A** hADSCs cells were treated with NR for 24 h, and acidic extracts were obtained to measure the total intracellular NAD+ content. **B** Screening for the optimal NR concentration in treating hADSCs. CCK-8 assay was conducted to test the viability of hADSCs after treatment with different dosages of NR. **C** The mRNA expression levels of COL2A1, SOX9 and ACAN in different groups of chondrogenesis-induced pellets were detected by qPCR. D The protein expression levels of COL2A1, SOX9, ACAN, COL10A1, RUNX2, MMP13, and COL1A1 in different groups of chondrogenesis-induced pellets. **E** Western blot analysis of proteins with densitometry corresponding to Fig. 1D. **F** Immunohistochemistry for COL2A1 and ACAN in pellets from different chondrogenesis induction groups. Mean density (IOD/Area) was used to quantify the COL2A1 and ACAN contents in the chondrogenic pellet. G and H, Immunofluorescence images of hADSCs undergoing chondrogenic induction for 1 week treated with or without NR, stained for COL2A1(G) and SOX9(H). Scale bar:100 μm. I Safranin O and Alcian Blue staining results of chondrogenesis induced pellet and corresponding. Quantification of the Safranin O and Alcian Blue staining was presented as the relative integrated density per area. Images were inverted for better visualization of the bar graph. Data are presented as means ± SDs (n = 3 independent biological replicate experiments). *p < 0.05, **p < 0.01, ***p < 0.001 (two-tailed Student’s t-test)

The hADSCs were obtained from liposuction in healthy humans. According to a flow cytometric analysis, the hADSCs were positive for CD73, CD90, CD105 and negative for the endothelial markers CD45 and HLA-DR (Figure S1A). the multiple differentiation ability of hADSCs was confirmed by induced differentiation into adipocytes, osteocytes, and chondrocytes, as indicated by Oil Red O, Alizarin Red, and Alcian Blue staining (Figure S1B). The optimal and safe concentration of NR was determined based on the NAD^+^ content detection and cell viability assay in hADSCs. NR treatment dose-dependently increased intracellular NAD^+^ levels in hADSCs (Fig. 1A), with maximal effects at concentrations upon 400 µM. In addition, after 24 h NR treatment, differences in cell viability were observed among the different concentrations of NR by CCK-8 assay, which showed 200 µM NR was optimal (Fig. 1B). Therefore, we choose 200 µM NR for subsequent experiments.

Subsequently, the effect of NR on chondrogenic differentiation of hADSCs was determined by adding NR to the chondrogenic inducing reagent. The induced-cartilage pellets were obtained after 21 days of chondrogenic differentiation, and mRNA and proteins were extracted. NR treatment significantly enhanced the expression levels of cartilage markers, COL2A1, SOX9, and ACAN on both mRNA and protein levels (Fig. 1C, D and E). Meanwhile, NR treatment significantly suppressed the expression of hypertrophic and fibrocartilage markers, including COL10A1, RUNX2, MMP13, and COL1A1(Fig. 1D and E), indicating that NR might promote stable hyaline cartilage formation. In addition, immunohistochemistry analyses showed that NR treatment could up-regulate the expression levels of COL2A1 and ACAN in chondrogenesis induced pellets (Fig. 1F). The increased levels of COL2A1 and SOX9 was also observed after 1 week NR treatment in two-dimensional culture condition, which was confirmed by Immunofluorescence staining (Fig. 1G and H). Furthermore, hADSCs treated with NR exhibited more intense Safranin O stain and Alcian Blue stain than untreated group after 21 days of induction (Fig. 1I). This indicates a higher potential for cartilage formation. These results illustrate that NR promoted chondrogenic differentiation of hADSCs.

### NR treatment activates TGF-β/SMAD2/3 pathway

**Fig. 2:**
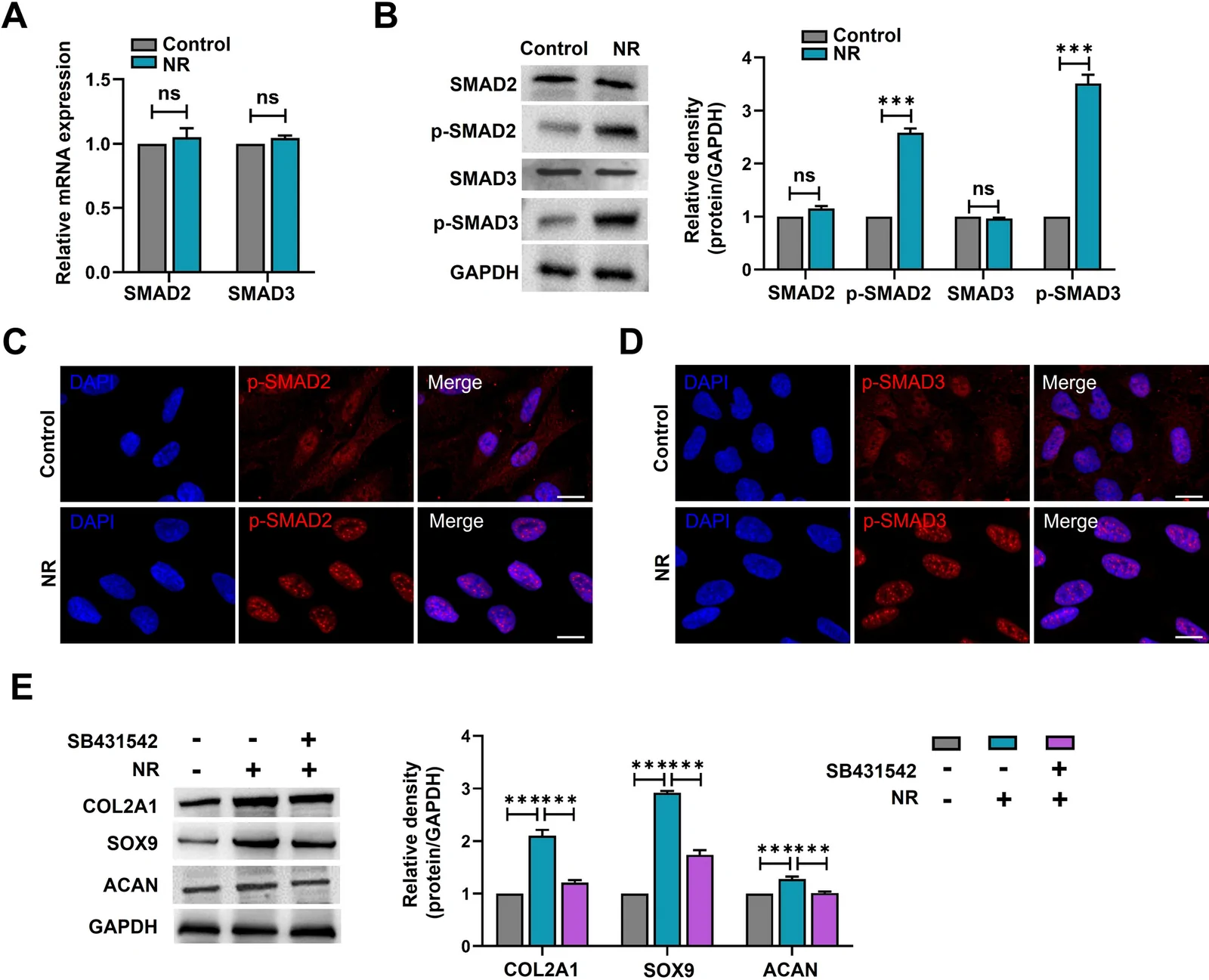
NR activates TGF-β/SMAD2/3 pathway. **A** The mRNA expression levels of SMAD2 and SMAD3 in the NR treatment group and the corresponding control group. **B** The protein expression levels of SMAD2, SMAD3, p- SMAD2 and p-SMAD3 in the NR treatment group and the corresponding control group. Western blot analysis was performed with densitometry. **C** and **D** Immunofluorescence of p-SMAD2 (**C**) and p-SMAD3(D) in hADSCs treated with NR for 7 days (scale bars: 10 μm). **E** hADSCs were untreated or treated with NR in presence or absence of the SMAD2/3 inhibitor (SB431542) for 24 h. The protein levels of COL2A1 ACAN and SOX9 were evaluated. Data are presented as means ± SDs (n = 3 independent biological replicate experiments). *p < 0.05, **p < 0.01, ***p < 0.001 (two-tailed Student’s t-test in A and B; two-way ANOVA in E)

Subsequently, we explored the possible mechanism accounting for the role of NR on chondrogenic differentiation in hADSCs. TGF-β has been extensively studied for its role in inducing chondrogenesis, with the TGF-β/SMAD2/3 signaling pathway recognized as the primary mechanism involved. Therefore, we examined whether NR could modulate the TGF-β/SMAD2/3 signaling pathway. Verification of the key molecules of the TGF-β signaling pathway, including SMAD2 and SMAD3, was performed. We found that both mRNA and protein expression levels of SMAD2/3 was not significantly increased in the NR treated group (Fig. 2A, B). However, the phosphorylation levels of SMAD2/3 were significant enhanced in the NR treated group (Fig. 2B). Moreover, immunofluorescence staining analysis revealed enhanced nuclear localization of p-SMAD2/3 following NR treatment (Fig. 2C and D). To further explore the mechanism, antagonists of TGF-β/SMAD2/3 pathway, SB431542 (10 μm SMAD2/3 inhibitor) was employed. Compared with the control group, NR treatment obviously enhanced the expression of cartilage matrix components (COL2A1, ACAN, and SOX9) revealed that NR promoted chondrogenic differentiation, while SB431542 inhibited these changes (Fig. 2E). These results indicate that the TGF-β/SMAD2/3 signaling pathway was activated during the chondrogenic differentiation of hADSCs induced with NR. Furthermore, activation of the TGF-β/SMAD2/3 signaling pathway represents a crucial mechanism through which NR enhances the chondrogenic differentiation of hADSCs.

### NR promotes SIRT1 during chondrogenic differentiation of hADSCs

**Fig. 3:**
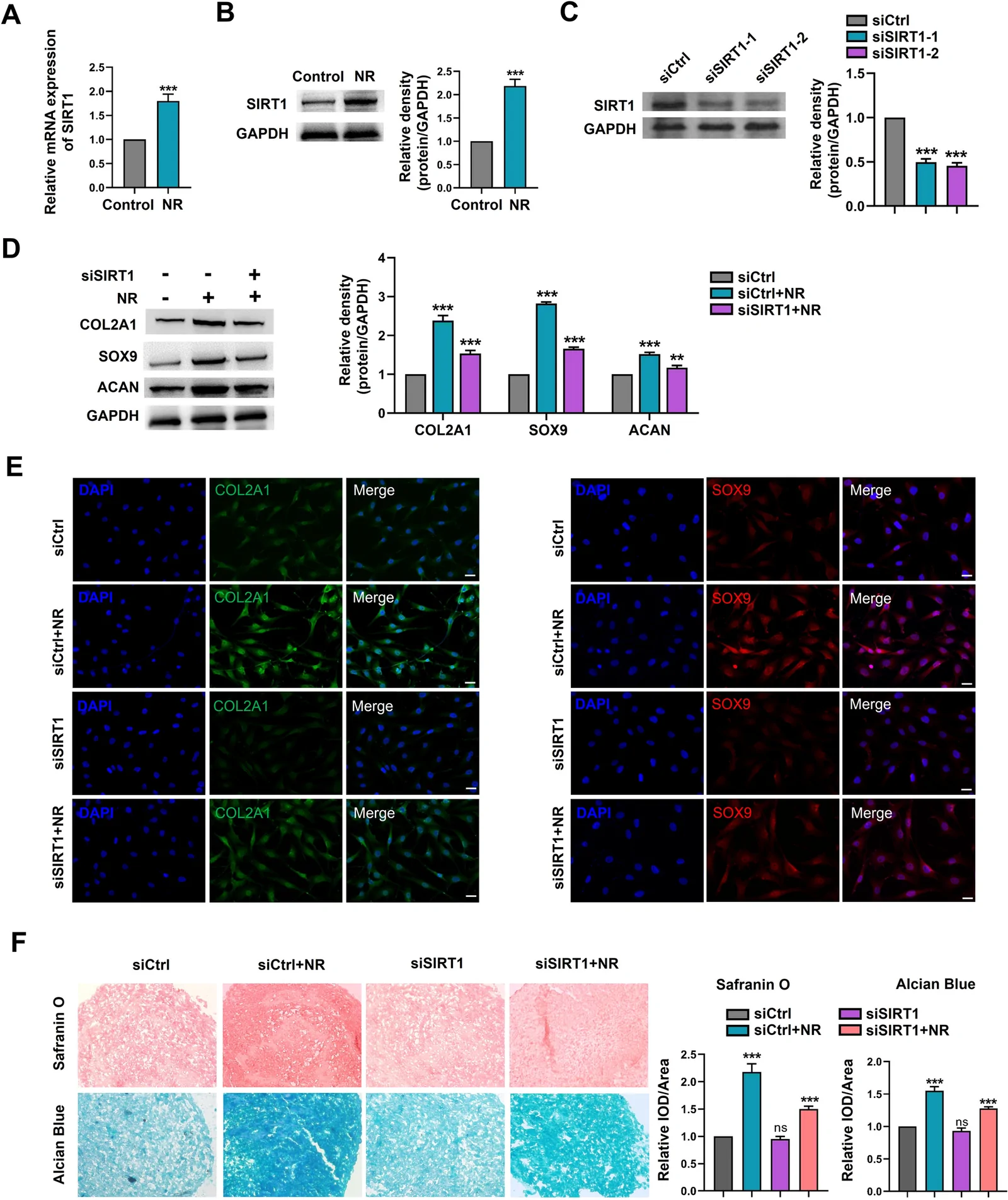
NR promotes chondrogenic differentiation of hADSCs by upregulating SIRT1. **A** The mRNA expression level of SIRT1 in hADSCs that were treated with or without NR for 7 days. **B** The protein expression level of SIRT1 in hADSCs that were treated with or without NR for 7 days. **C** The protein expression level of SIRT1 in the indicated cells. D. The protein levels of COL2A1, SOX9 and ACAN in the indicated cells. **E** Immunofluorescence of COL2A1 and ACAN in indicated cells (scale bars: 100 μm). **F** Safranin O and Alcian Blue staining of hADSCs-induced cartilage tissue on 7 days in different groups. Quantification of the Safranin O and Alcian Blue staining was presented as the relative integrated density per area. Data are presented as means ± SDs (n = 3 independent biological replicate experiments). *p < 0.05, **p < 0.01, ***p < 0.001 (two-tailed Student’s t-test in A and B; two-way ANOVA in C, D and F)

NR exhibits a protective effect in various diseases is primarily attributed to its role in enhancing NAD^+^ levels. Sirtuins as a family of proteins whose basal activity can be largely modulated by NAD^+^ availability. Among them, SIRT1 is widely recognized as a NAD^+^-dependent deacetylase. Therefore, we wonder whether NR influences SIRT1 during the chondrogenic differentiation of hADSCs. Interestingly, NR treatment significantly upregulated the mRNA and protein level of SIRT1 (Fig. 3A and B). To investigate the role of SIRT1 in NR-induced chondrogenic differentiation, we generated SIRT1 knockdown cell lines (Fig. 3C). The downregulation of SIRT1 attenuated the enhancing effects of NR on the expression of COL2A1, SOX9, and ACAN (Fig. 3D). Moreover, Immunofluorescence staining results indicated that compared with the significant increase in the levels of cartilage marker proteins observed in wild-type cells after NR treatment, the changes in protein expression before and after NR supplementation in SIRT1 knockdown cells were less obvious (Fig. 3E). Furthermore, compared with the control cells, the NR-enhanced Safranin O and Alcian Blue staining intensities were both reduced in the SIRT1 knockdown cells (Fig. 3F). These findings showed that SIRT1 knockdown suppresses NR-mediated promotion of chondrogenic differentiation. Therefore, the promotion of chondrogenic differentiation of hADSCs by NR is associated with its regulation of SIRT1.

### NR activates the TGF-β/SMAD2/3 pathway by enhancing SIRT1 activity to deacetylate SMAD7

**Fig. 4:**
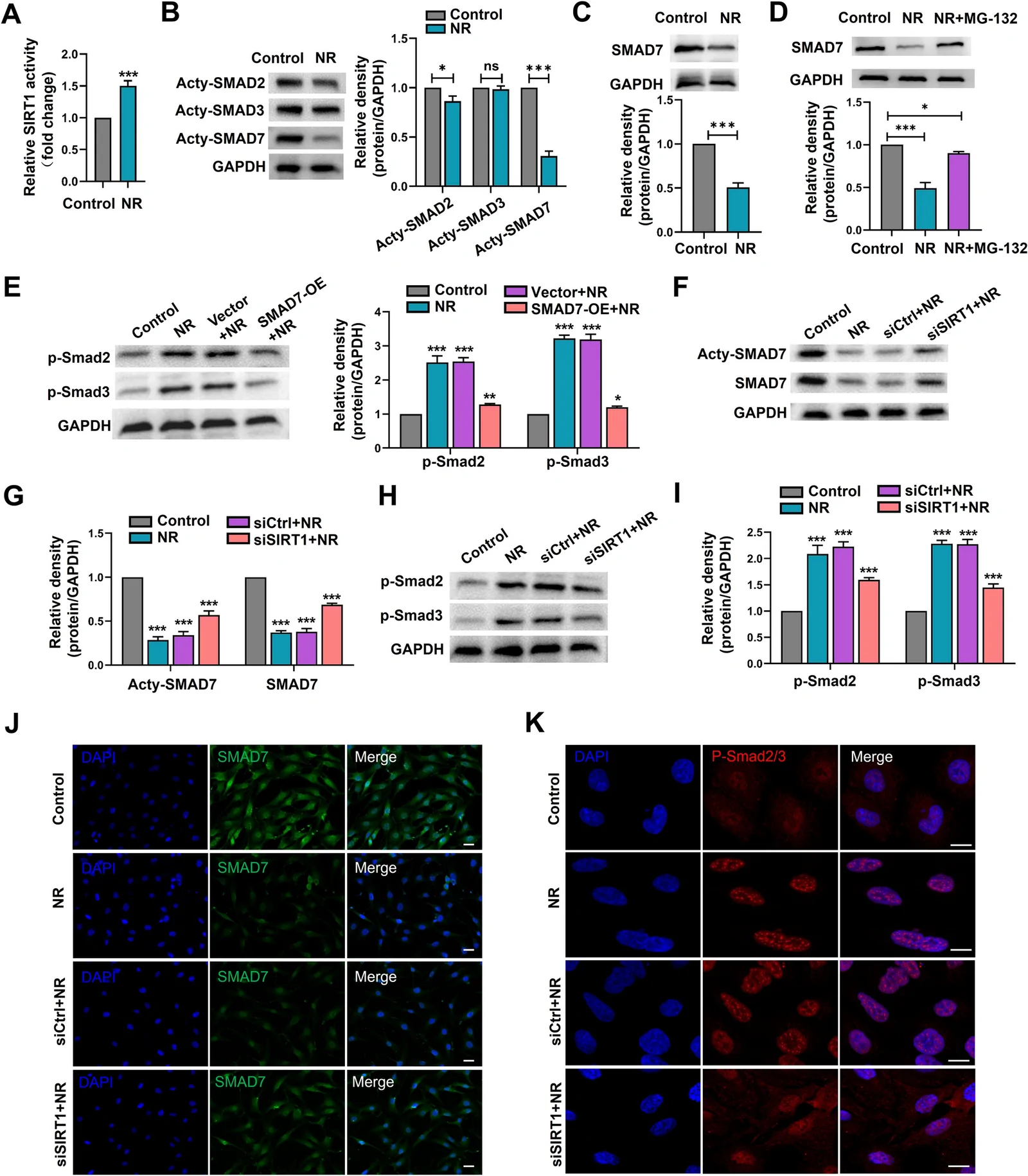
NR deacetylates SMAD7 and modulates the phosphorylation of SMAD2/3. **A** The relative SIRT1 deacetylase activity in hADSCs treated with or without NR was measured using a SIRT1 Activity Assay Kit (ab156065, Abcam, US) according to the manufacturer’s instructions. **B** The acetylation levels of SMAD2, SMAD3, and SMAD7 were determined in hADSCs treated with or without NR for 7 days. **C** The protein level of SMAD7 in hADSCs treated with or without NR for 7 days. **D** The protein level of SMAD7 in hADSCs treated with or without NR in presence or absence of MG-132 for 6 h. E, H The protein levels of p-SMAD2 and p-SMAD3 in the indicated cells that were treated with or without NR for 7 days. F The protein level of SMAD7 and its acetylation level of SMAD7 in the indicated cells that were treated with or without NR for 7 days. **G**, **I** Western blot analysis of the proteins with densitometry was performed corresponding to Fig. 4F and **H**. **J**, **K** Immunofluorescence of SMAD7(J) and p-SMAD2/3(K) in indicated cells treated with or without NR for 7 days. Data are presented as means ± SDs (n = 3 independent biological replicate experiments). *p < 0.05, **p < 0.01, ***p < 0.001 (two-tailed Student’s t-test in **A**, **B** and **C**; two-way ANOVA in **D**, **E**, **G** and **I**)

SIRT1 was reported as a NAD-dependent deacetylase. The ability of NR to increase the intracellular level of NAD^+^ has led us to hypothesis that, in addition to altering the level of SIRT1, it may also enhance the activity of SIRT1. Indeed, NR enhanced the deacetylase activity of SIRT1(Fig. 4A). In addition, we wonder whether the enhancement of SIRT1 activity by NR is related to its promotion of the SMAD signaling pathway. To address our hypothesis, the acetylation level of primary TGF-β/SMAD2/3 pathway components were examined in the presence of NR treatment. NR reduced the acetylation of SMAD2 and SMAD3, but not obvious (Fig. 4B). Interestingly, we found that NR significantly reduced the acetylation of SMAD7 (Fig. 4B). As deacetylation is crucial for protein stability, and a previous study has demonstrated that SIRT1‑mediated deacetylation facilitates Smad7 proteasomal degradation [21]. We proposed that SIRT1 deacetylates SMAD7 and promotes its degradation in our system. Indeed, NR treatment significantly decreased the protein level of SMAD7 as compared to control (Fig. 4C). In addition, the treatment with proteasome Inhibitor MG-132 prevented NR-induced Smad7 downregulation (Fig. 4D). SMAD7 was reported inhibits TGF-β signaling by inhibiting SMAD2/3 phosphorylation and nuclear localization. Therefore, we proposed that NR induces promotion of SMAD2/3 phosphorylation may be associated with the inhibition of SMAD7, thereby attenuating the inhibitory effect of SMAD7 on SMAD2/3 signaling pathway. Indeed, overexpression of SMAD7 remarkably abolished the NR-mediated upregulation of p-SMAD2/3 (Fig. 4E). Moreover, NR-promoted deacetylation of SMAD7 is in a SIRT1-dependent manner. Knockdown of SIRT1 inhibited the effect of NR on acetylation of SMAD7 expression and SMAD7 stability (Fig. 4F and G). Meanwhile, Knockdown of SIRT1 inhibited the effect of NR on promoted p-SMAD2/3 level (Fig. 4H and I). Furthermore, Immunofluorescence results confirmed that SIRT1 knockdown significantly restored SMAD7 level (Fig. 4J) and reduced p-SMAD2/3 expression and its nuclear localization (Fig. 4K) upon NR treatment under chondrogenic inducing. This suggested that NR enhanced the activation of SIRT1, which deacetylates SMAD7 and reduced SMAD7 protein stability, results in enhanced phosphorylation of SMAD2/3 and promoted SMAD2/3 pathway during chondrogenic differentiation of hADSCs.

### SIRT1-dependent deacetylation of SMAD7 is a key factor in NR-induced chondrogenesis

**Fig. 5:**
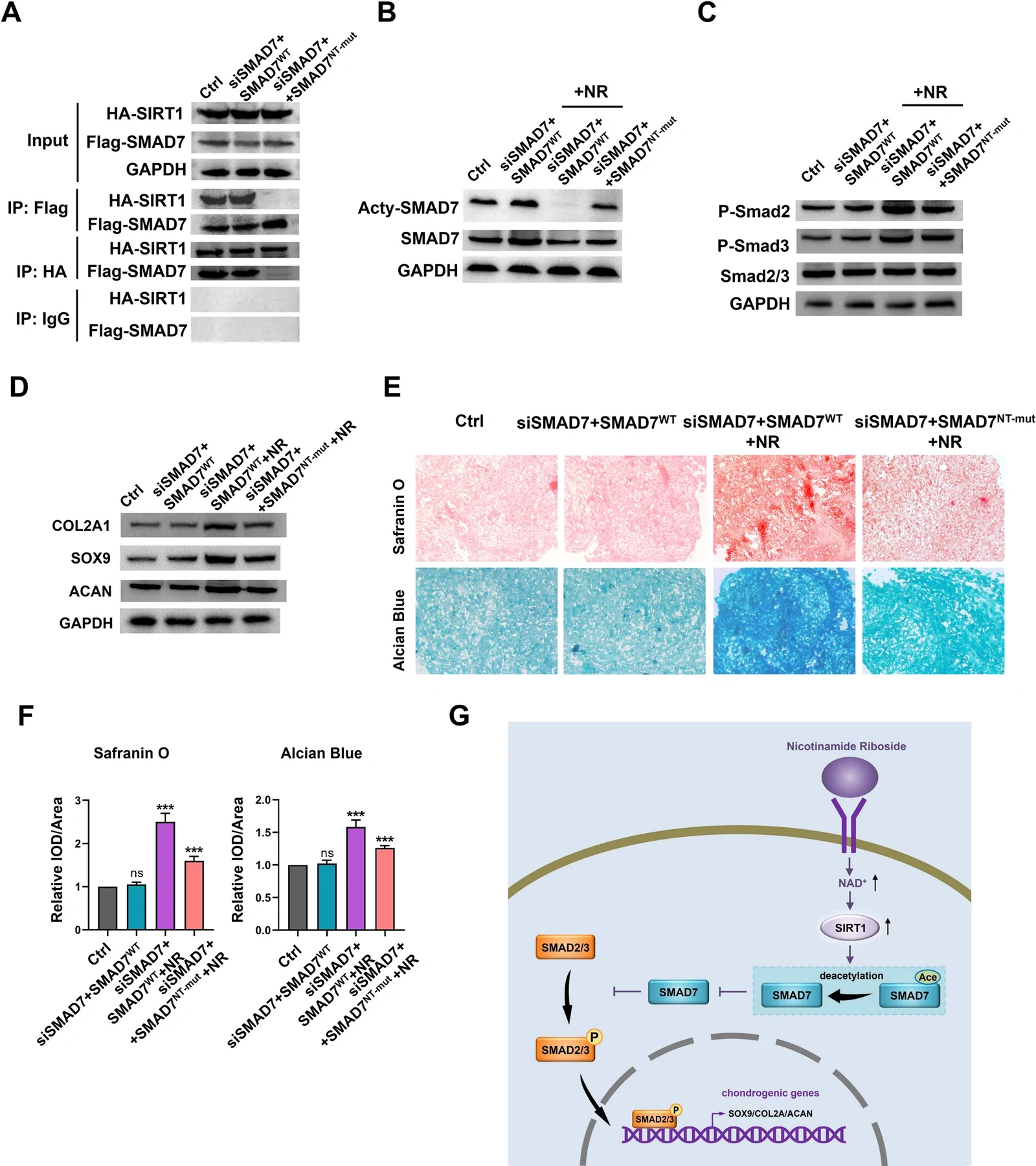
Interaction of SIRT1 and SMAD7 is important for NR-induced enhancement of chondrogenic differentiation in hADSCs. **A** Co-IP assay was performed in the indicated cells. Immunoprecipitation was conducted using HA-tagged SIRT1, Flag-tagged SMAD7, or IgG antibody in the indicated cells subjected to NR treatment, followed by immunoblotting for HA and Flag. **B** The protein levels of SMAD7 and the acetylation level of SMAD7 in the indicated cells treated with or without NR for 7 days. **C** The protein levels of SMAD2/3, p-SMAD2 and p-SMAD3 in the indicated cells treated with or without NR for 7 days. **D** The Protein levels of COL2A1, SOX9 and ACAN in the indicated cells treated with or without NR for 7 days. E Safranin O and Alcian Blue staining of hADSCs-induced cartilage tissue on 7th day in different groups. F Quantification of the Safranin O and Alcian Blue staining in Figure **E** was presented as the relative integrated density per area. G Schematic representation of NR enhanced NAD-dependent deacetylase SIRT1, promoting the deacetylation of SMAD7, facilitating an increase in p-SMAD2/3 level and TGF-β/SMAD2/3 pathway, thereby enhancing chondrogenic differentiation of hADSCs. Data are presented as means ± SDs (n = 3 independent biological replicate experiments). *p < 0.05, **p < 0.01, ***p < 0.001 (two-way ANOVA in **F**)

Subsequently, we investigated the importance of SIRT1-dependent deacetylation of SMAD7 accounting for the role of NR on chondrogenic differentiation in hADSCs. SIRT1 was reported interacted with SAMD7 via motifs located in the N-terminal of SMAD7 [21]. Therefore, we generated a SMAD7 mutant, referred to as SMAD7^NTmut^, to delete the N-terminal motif (residues 1 to 589), in order to disrupt interaction between SIRT1 and SMAD7. The results showed that SIRT1 interacts with wild-type (WT) SMAD7^WT^ but not with SMAD7^NT−mut^ (Fig. 5A). Consequently, the acetylation level of SMAD7 was significantly reduced in siSMAD7 + SMAD7^WT^ cells upon NR treatment, and this effect of NR was no longer significant in siSMAD7 + SMAD7^NT−mut^ cells (Fig. 5B). Moreover, NR induced enhancement of p-SAMD2/3 was also not observed in siSMAD7 + SMAD7^NT−mut^ cells (Fig. 5C), indicating that the loss of interaction between SIRT1 and SMAD7 results in the elimination of NR-induced SMAD2/3 activation. Furthermore, the NR-induced enhancement of chondrogenic differentiation in hADSCs was attenuated in cells with impaired SIRT1-mediated deacetylation of SMAD7 (Fig. 5D and E). These results demonstrated that SIRT1-dependent deacetylation of SMAD7 is required for the NR-induced enhancement of chondrogenic differentiation in hADSCs.

## Discussion

The application of MSCs in cartilage regeneration represents a promising therapeutic strategy for promoting cartilage repair, addressing cartilage defects, and managing cartilage-related diseases. Nevertheless, several challenges remain in clinical applications, particularly regarding the efficiency of chondrogenic differentiation. Our study has identified nicotinamide riboside (NR) capable of significantly enhancing the chondrogenic differentiation efficiency of hADSCs. The expression of chondrogenesis markers was significantly elevated by the addition of NR during chondrogenesis induction. Mechanistically, we demonstrated that NR activated the TGF-β/SMAD2/3 pathway. Further investigation revealed that NR enhanced both the expression and activity of SIRT1, thereby promoting the deacetylation of SMAD7 and subsequent degradation. Finally, the downregulation of SMAD7 led to an increase in p-SMAD2/3 level and promoted its nuclear translocation, triggering the transcription of chondrocyte-related target genes, including COL2A1, ACAN, and SOX9 (Fig. 5G). Our study provides evidence that NR promotes chondrogenic differentiation of hADSCs and may be applied as a supplement for cartilage regeneration and repair.

Increasing NAD^+^ levels by NR have been detected in multi biological samples, such as yeast, cultured human cells and mammalian tissues. As a NAD^+^ booster, NR has been attracted provides protective benefits in multiple diseases, including neurodegenerative diseases, liver fibrosis and heart failure [25–27]. However, research on the effects of NR in cartilage-related diseases remains limited. Our study suggests that NR acts as an enhancer for the chondrogenic differentiation of hADSCs and may serve as a supplementary agent in therapeutic strategies to promote cartilage regeneration. As a small molecule, NR has been demonstrated to possess numerous advantages, including favorable metabolic characteristics, excellent safety and efficacy profiles, and oral bioavailability in humans, making it highly suitable for clinical therapeutic applications. The findings of this study further indicate the potential involvement of small molecules in the induction of chondrogenic differentiation, prompting speculation that additional small molecules may also can act as enhancers in stem cell chondrogenic differentiation. Given the excellent biocompatibility and greater clinical applicability of small molecules, the promotion of cartilage differentiation by small molecules deserves further research.

In this study, NR was demonstrated that enhanced chondrogenic differentiation of hADSCs by regulating SIRT1-mediated deacetylation of SMAD7 and subsequently activating the TGF-β/SMAD2/3 signaling pathway. We found that the influence of NR on the acetylation of SMAD7 is significantly greater than its effect on the acetylation of SMAD2 or SMAD3, indicating that the impact of NR on acetylation levels varies across different genes. In addition, SIRT1, SIRT2 and SIRT3 are all NAD^+^-dependent deacetylases. However, treatment with NR in induced hADSCs upregulated the expression of SIRT1, without affecting the other two. It would be valuable to further investigate the mechanisms underlying the inconsistent regulatory effects caused by NR. Certainly, we do not exclude the possibility that NR may participate in the regulation of chondrogenic differentiation through additional pathways, which warrants further investigation in future studies.

A limitation of this study is that all experiments were conducted in vitro, and the results have not been validated in vivo. Although the current findings provide mechanistic insights into how NR enhances chondrogenic differentiation of hADSCs by inducing SIRT1-mediated deacetylation of SMAD7 and activation of TGF-β/SMAD2/3 pathway, their in vivo significance remains to be established in appropriate animal models. Further in vivo studies are required to confirm our observations and evaluate their physiological relevance.

In conclusion, our findings demonstrate that NR enhanced chondrogenic differentiation of hADSCs by regulating SIRT1-mediated deacetylation of SMAD7, thereby influencing phosphorylation of SMAD2/3 and TGF-β/SMAD2/3 pathway. Our study provides evidence that NR may serve as a promising supplement for MSC-based cartilage regeneration and repair therapies.

## Electronic Supplementary Material

Below is the link to the electronic supplementary material.

Supplementary Material 1.

## Data Availability

No datasets were generated or analysed during the current study.
