# Supplementary material for: Nicotinamide riboside enhances chondrogenic differentiation of human adipose stem cells via inducing SIRT1-mediated deacetylation of SMAD7 and activation of TGF-β/SMAD2/3 pathway
Source: Stem Cell Res Ther. 2026 Aug 8;17:410. doi: 10.1186/s13287-026-05226-8 (PMC13737904; doi:10.1186/s13287-026-05226-8)
Supplement: Supplementary file 1 [file 13287_2026_5226_MOESM1_ESM.docx]

**Supplementary information**

**Table S1. Primer sequences for quantitative** **PCR, Related to Figure 1 to Figure 4.**

| Target Gene | Primers (5’-3’) |
| --- | --- |
| β-Actin | F: CATGTACGTTGCTATCCAGGC |
|  | R: CTCCTTAATGTCACGCACGAT |
| COL2A1 | F: |
|  | R: |
| SOX9 | F: |
|  | R: |
| ACAN | F: |
|  | R: |
| SMAD2 | F: |
|  | R: |
| SMAD3 | F: |
|  | R: |
| SIRT1 | F: |
|  | R: |

F, Forward; R, Reverse

**Figure S1 Characterization of hADSCs.**

**A** Flow cytometry of hADSC surface markersCD73, CD90, CD105, CD146, CD34, and CD45. **B**. Adipogenic, osteogenic, and chondrogenic differentiation was evaluated by Oil-Red-O, Alizarin Red and Alcian Blue staining.
